# Change in brain glucose metabolism and cognition in amnestic MCI patients in relation to amyloid accumulation: A 3-year follow-up study

**DOI:** 10.1016/j.nbas.2026.100165

**Published:** 2026-08-06

**Authors:** Elina Rauhala, Jarkko Johansson, Mira Karrasch, Olli Eskola, Tuula Tolvanen, Johan Rajander, Juha O. Rinne

**Affiliations:** a Clinical Neurosciences, Faculty of Medicine, University of Turku and Neurocenter, Turku University Hospital, Turku, Finland; b Turku PET Centre, University of Turku, Turku University Hospital, Turku, Finland; c Department of Diagnostics and Intervention, Umeå University, Umeå, Sweden; d Department of Psychology, Åbo Akademi University, Turku, Finland; e Radiopharmaceutical Chemistry Laboratory, Turku PET Centre, University of Turku, Turku, Finland; f Department of Medical Physics, Turku University Hospital, Turku, Finland; g Accelerator Laboratory, Turku PET Centre, Åbo Akademi University, Turku, Finland

**Keywords:** Fluorodeoxyglucose, Positron emission tomography, Mild cognitive impairment, Alzheimer, Amyloid

## Abstract

Our aim was to investigate the change in brain glucose metabolism with [^18^F]fluorodeoxyglucose positron emission tomography ([^18^F]FDG PET) in amyloid beta positive and negative amnestic mild cognitive impairment (aMCI) patients. We investigated associations between change in [^18^F]FDG uptake and cognitive functioning in aMCI patients over up to 3 years. Twenty patients underwent [^18^F]FDG PET (15 men and 5 women, mean age 73.0 years, SD 6.3 at baseline) within a short time interval from [^18^F]Flutemetamol PET. The patients were classified as amyloid positive vs. negative based on the amyloid PET marker [^18^F]Flutemetamol uptake. Patients were examined with brain MRI and cognitive tests at baseline and after 3-year follow-up or earlier if the patient had converted to probable Alzheimer's disease. [^18^F]FDG data was analysed with automated region-of-interest analysis and voxel-based statistical parametric mapping (SPM). [^18^F]FDG uptake nor change of [^18^F]FDG uptake over time differed between amyloid positive and negative groups. However, the amyloid positive patients exhibited significant decline in MMSE scores over three years while the amyloid negative patients maintained stable MMSE level over time. The amyloid positive group had poorer episodic memory scores at baseline and follow-up, and significantly slower processing speed at follow-up as well as significantly more negative change over time in language functions compared to amyloid negative patients. Moreover, we found that [^18^F]FDG uptake was positively associated with cognition in the whole aMCI patient group. Together, our results suggest that brain glucose metabolism in aMCI is independent of amyloid pathology, and probably reflects other brain degenerative processes.

## 1 Background

In amnestic Mild Cognitive Impairment (aMCI), a substantial proportion of patients clinically progress to Alzheimer's disease (AD). In MCI and AD, a common pathological sign is decline in glucose metabolism which can be assessed by [^18^F]fluorodeoxyglucose positron emission tomography ([^18^F]FDG PET) [1]. Cerebral glucose hypometabolism has been suggested as one of the early features in the pathogenesis of AD [2], [3], [4], and [^18^F]FDG PET measuring glucose metabolism has been shown as an impactful biomarker in aMCI [5], [6], [7], [8]. Moreover, elevated brain amyloid load has been associated with cognitive decline in MCI patients which has been shown in follow-up studies assessed with [^18^F]Flutemetamol [9], [10], [11] and [^11^C]PIB PET [12], [13], [14], [15].

[^18^F]FDG hypometabolism has been commonly observed in temporoparietal, occipital and posterior cingulate cortices as well as the precuneus of AD patients compared to healthy controls [4], [5], [16]. Widespread hypometabolism was also found in MCI patients who later converted to AD compared to non-converters [7], [17]. In MCI patients hypometabolism has been observed specifically in the hippocampus [6], [18] and in the inferior parietal lobe [6]. However, [^18^F]FDG PET studies have also shown controversial findings regarding glucose metabolism in MCI patients. Instead of decrease in [^18^F]FDG uptake, also higher or preserved brain metabolism has been reported in cross-sectional studies [17], [19], [20], [21].

Longitudinal [^18^F]FDG PET studies have been used to monitor metabolic change in aMCI patients [12], [22] and its association to cognitive performance [23], [24], [25]. In amyloid positive aMCI patients, a faster decline in parietal glucose metabolism over two years was found, and it was associated with a decline in global cognition and episodic memory functions [23]. Contrary to this, Dubois et al. [26] reported no differences between amyloid positive and negative MCI patients in any of the most used cognitive tests at 12 months or 24 months follow-up in early-onset MCI patients. Furthermore, parallel negative changes in MMSE and glucose metabolism have been reported in a 2-year follow-up in stable MCI and prodromal AD patients though the decline in MMSE in stable MCI group was minimal [27]. Also, brain amyloid accumulation was not taken into account in that study. Accordingly, a longitudinal study of over 4 years showed that decrease in brain glucose metabolism was associated with cognitive decline in aMCI patients regardless of amyloid status, while glucose metabolism in controls remained fairly constant over time [25]. In subjects with subtle cognitive deficits, a correlation between increased glucose metabolism and worse verbal memory performance has been found [28]. In the same patients, amyloid accumulation was associated with subtle cognitive deficits before the emergence of hypometabolism. As clinical symptoms progress toward AD, hypometabolism and its negative consequences for cognition may become the prevailing pattern of glucose metabolism. Yet, the precise longitudinal characterization of amyloid-metabolic-cognitive associations within early stage aMCI subjects has remained elusive.

The aim of this study was to examine the change in brain glucose metabolism and its relation to cognition in amyloid negative and positive aMCI patients over a 3-year follow-up. Earlier studies have found both faster decline or no differences in glucose metabolism and cognition in amyloid positive versus negative patients. In this study we investigated specifically aMCI patients, with [^18^F]Flutemetamol, [^18^F]FDG PET and MRI scans, and cognitive tests.

## 2 Material and methods

### 2.1 Subjects

Demographics of the patients are shown in Table 1. Altogether 34 patients (23 men and 11 women) were examined with [^18^F]Flutemetamol PET, which we have reported previously [10]. Twenty subjects of those underwent also [^18^F]FDG PET (15 men and 5 women) within a short time interval from the [^18^F]Flutemetamol PET. Thus, the present study represents a longitudinal [^18^F]FDG PET subanalysis of the previously reported [^18^F]Flutemetamol aMCI cohort [10], restricted to patients with available [^18^F]FDG PET, cognitive follow-up, and baseline amyloid-PET classification. The mean age of those 20 patients was 73.0 ± 6.3 (mean ± SD) at baseline. All patients met the criteria of aMCI [29]. All patients gave their written informed consent, which was obtained according to the requirements of the Declaration of Helsinki. The study protocol was approved by the Ethics Committee of Hospital District of South-West Finland.

**Table 1:** Demographics of the study participants. Two-sample *t*-tests were conducted to assess whether amyloid positive and negative patients differed in terms of mean age and education.

	All	Amyloid positive	Amyloid negative
Patients at baseline	*N* = 20	*N* = 9	*N* = 11
Mean age at baseline (years)	73.0 ± 6.3	75.8 ± 4.5	70.8 ± 6.8
Age range at baseline (years)	60–83	68–82	60–83
Mean education (years)	12.6 ± 2.6	13.4 ± 2.6	11.9 ± 2.4
Range in education (years)	9–16	9–16	9–16
Males (%)	*N* = 15 (75.0)	*N* = 7 (77.8)	*N* = 8 (72.7)
Females (%)	*N* = 5 (25.0)	N = 2 (22.2)	*N* = 3 (27.3)

No significant group differences were observed.

The patients were assessed with [^18^F]FDG PET, and [^18^F]Flutemetamol PET scans, brain MRI and cognitive tests. The cognitive tests were administered yearly until clinical progression to probable Alzheimer's disease, or until the end of the 3-year follow-up. Probable Alzheimer's disease was diagnosed when the patients fulfilled the National Institute of Neurological and Communicative Disorders and Stroke-Alzheimer's Disease and Related Disorders Association criteria as well as DSM-IV criteria for dementia of the Alzheimer type. MRI and [^18^F]FDG PET were repeated after the subject had progressed to probable Alzheimer's disease or after 3 years had elapsed from the first [^18^F]FDG PET scan. The term probable Alzheimer's disease is used here to denote the clinical diagnostic category applied in the study protocol, supported by the available amyloid-PET information, but not full biological Alzheimer's disease characterization according to the ATN framework because tau biomarkers were not available [30]. Patients were classified as amyloid-positive or amyloid-negative based on the baseline [^18^F]Flutemetamol scan using a quantitative composite cortical uptake ratio. Amyloid positivity was defined as a composite cortical [18F]Flutemetamol uptake ratio > 1.4, according to the previously established threshold [31]. The classification used in the present analyses was therefore based on the quantitative uptake ratio rather than visual read. ApoE genotype data were not available for this cohort. Patients with amyloid positive and negative [^18^F]Flutemetamol scans did not differ statistically regarding education and age at baseline (Table 1). Cognitive test performances of the two groups at baseline and follow-up are presented in Table 2.

**Table 2:** Cognitive test results stratified by test time and amyloid status. Mean (±SD) cognitive scores are shown in each patient group and time point. Paired t-tests were conducted to assess the significance of change over time (delta), and two-sample t-tests were conducted to assess the group difference in level and change across the amyloid positive and negative patients.

	All	Amyloid Positive	Amyloid Negative	Group Difference
MMSE baseline	26.9 ± 1.7 (N = 20)	26.7 ± 1.9 (N = 9)	27.1 ± 1.5 (N = 11)	t(18) = −0.55
MMSE followup	25.6 ± 3.2 (*N* = 18)	23.6 ± 2.8 (N = 7)	26.8 ± 2.9 (N = 11)	**t(16)** **=** −2.33*
MMSE delta	−1.4 ± 3.1.	**−3.1** **±** 2.5*	−0.3 ± 3.0	t(16) = −2.07.
Episodic memory baseline	0.1 ± 0.8 (N = 20)	−0.4 ± 0.5 (N = 9)	0.6 ± 0.8 (N = 11)	**t(18)** **=** −3.30**
Episodic memory followup	−0.1 ± 0.9 (N = 18)	−0.8 ± 0.7 (N = 7)	0.3 ± 0.7 (N = 11)	**t(16)** **=** −3.38**
Episodic memory delta	**−0.3** **±** 0.6*	−0.4 ± 0.7	−0.2 ± 0.6	t(16) = −0.70
Executive functions baseline	0.2 ± 0.6 (N = 20)	0.1 ± 0.6 (N = 9)	0.2 ± 0.6 (N = 11)	t(18) = −0.15
Executive functions followup	−0.2 ± 0.6 (N = 20)	−0.3 ± 0.7 (N = 9)	−0.1 ± 0.6 (N = 11)	t(18) = −0.51
Executive functions delta	**−0.4** **±** 0.6*	−0.4 ± 0.6.	−0.3 ± 0.7	t(18) = −0.37
Processing speed baseline	0.0 ± 0.9 (N = 20)	−0.2 ± 1.0 (N = 9)	0.2 ± 0.7 (N = 11)	t(18) = −1.21
Processing speed followup	−0.0 ± 0.7 (N = 20)	−0.4 ± 0.6 (N = 9)	0.3 ± 0.7 (N = 11)	**t(18)** **=** −2.11*
Processing speed delta	−0.1 ± 0.6	−0.2 ± 0.8	0.0 ± 0.5	t(18) = −0.62
Language functions baseline	−0.1 ± 0.7 (N = 20)	−0.1 ± 0.8 (N = 9)	−0.1 ± 0.7 (N = 11)	t(18) = −0.13
Language functions followup	0.1 ± 0.8 (N = 18)	−0.4 ± 1.0 (N = 7)	0.4 ± 0.6 (N = 11)	t(16) = −1.91.
Language functions delta	0.2 ± 0.6	−0.2 ± 0.6	**0.4** **±** 0.5*	**t(16)** **=** −2.32*

* *p* < 0.05, ** *p* < 0.01. MMSE = Mini-mental state examination.

### 2.2 Cognitive tests

The cognitive performance of patients was comprehensively assessed once a year or when the aMCI patient had converted to probable AD. Here, the scores from the tests conducted at baseline and follow-up closest in time to the [^18^F]Flutemetamol PET scan were included in the analysis. The test battery included Mini-Mental State Examination (MMSE) as a measure for global cognitive functioning. The following cognitive tests were administered: Digit Span Backward and Digit Symbol Substitution from WAIS-R (Wechsler, 1992), Logical memory I and II from WMS-R [32], [33], COWAT S-Fluency [34], Trail Making Test (TMT-A and TMT—B) [35], Category fluency, naming and word list delayed recall from CERAD [36], as well as the Stroop Task [37].

Domain-specific composite scores were calculated to reduce the number of cognitive variables and increase the reliability of the cognitive variables [38]. Because each cognitive domain composite was derived from several neuropsychological test variables measured in different units and scales, the individual test scores were standardized to z-scores before composite score calculation. Furthermore, inverted scores were used for timed tests (TMT-A, TMT—B, Stroop) so that higher score indicated better performance. In line with prior work, composite scores were calculated using the mean standardized score for each of the four cognitive domains. The Executive function (EF) composite score included TMT-B minus TMT-A, Stroop interference minus Stroop color naming task, Digit Span Backward, and S-Fluency [38]. The composite score for Processing speed included the TMT-A and Digit Symbol Substitution Tests. The Episodic memory composite score included Word List Delayed Recall and Logical Memory I and II. And finally, a Language functioning score included Category Fluency (animal) test and Naming task.

### 2.3 PET and MRI imaging

All patients underwent a [^18^F]FDG and [^18^F]Flutemetamol PET scans. They received approximately 205.2 ± 14.2 MBq (mean ± SD) of [^18^F]FDG and after injection underwent a 55-min dynamic scan. A late time frame (35–55 min) was used to calculate of [^18^F]FDG uptake ratio. The injected dose of [^18^F]Flutemetamol was approximately 187.8 ± 18.7 MBq (mean ± SD) and 90 min later a 30-min brain scan was performed.

The mean [^18^F]Flutemetamol and [^18^F]FDG scanning intervals between baseline and follow-up scans were 2.9 ± 0.4 years for both tracers. One amyloid positive patient's follow-up PET scans was excluded from the analysis due to error in patient positioning leading to inadequate cerebellar imaging precluding reliable determination of reference region.

Patients were examined with GE Discovery 690 (GE Healthcare, Waukesha, WI, US) PET scanner. GE Discovery 690 is a hybrid PET/CT scanner with the axial field of view of 15.7 cm and the patient port is 70.0 cm. Physical performance evaluations of the scanner have shown radial and tangential average spatial resolution of 4.70 mm FWHM and axial resolution of 4.74 mm FWHM [39].

All patients underwent brain MR imaging at baseline and follow-up using Philips 1.5 T Gyroscan Intera CV Nova Dual MR scanner (Philips Medical Systems, Best, The Netherlands). A head coil (Philips Medical Systems, Best, The Netherlands) was used in the measurement. Whole-brain T1-weighted three-dimensional fast field echo data with 1-mm isotropic voxels were acquired in the transverse plane (acquisition parameters: repetition time 25 ms, echo time 5.5. ms, flip angle 30°, field of view 256 × 256 mm) yielding at least 160 contiguous slices to cover the whole head. In addition, routine axial T2-weighted and coronal FLAIR images were obtained.

### 2.4 Image analyses

The procedures for the [^18^F]Flutemetamol analyses have been reported previously [10], and the [^18^F]FDG analyses followed for the most part the same procedures. Briefly, the [^18^F]Flutemetamol and [^18^F]FDG data were corrected for motion and registered to T1-weighted anatomical MR images using statistical parametric mapping software (SPM, version 8, Wellcome Institute). Spatial normalization to Montreal Neurological Institution (MNI) space was conducted on the basis of individual T1-weighted MRI using unified segmentation algorithm in SPM8. For [^18^F]Flutemetamol, an uptake ratio index was calculated in each voxel relative to the uptake in the cerebellar cortex, in line with our previous [^18^F]Flutemetamol analyses and the quantitative cut-off used for amyloid classification [31]. For [^18^F]FDG, the uptake ratio index was calculated relative to the pons region, which was selected as a reference region because of its relative preservation in Alzheimer-type neurodegeneration and its use in previous longitudinal FDG-PET studies of MCI and Alzheimer's disease. The pons region was manually delineated in the MNI space using an average T1-weighted image as the anatomical reference. In the present analyses average regional [^18^F]FDG uptake ratios were calculated using the Desikan-Killiany atlas [40], provided in FreeSurfer version 6.0. A composite cortical [^18^F]Flutemetamol uptake score was formed by combining the regions-of-interest (ROIs) of frontal, parietal and lateral temporal cortices and posterior cingulate, similar to our earlier investigations [41]. A composite cortical [^18^F]FDG consisted of precuneus, posterior cingulate and lateral temporal and parietal cortices which are known to be hypometabolic in AD [4], [5], [16], [24], [42]. To assess whether the use of this composite ROI could have diluted more regionally restricted metabolic effects, we additionally performed group difference analyses separately for each anatomical Desikan–Killiany cortical region (*n* = 42). These region-wise analyses were used as supplementary sensitivity analyses and are reported in Supplementary Table 1.

### 2.5 Statistical analyses

VOI-based statistical analyses were conducted using R (version 4.0.3). Descriptive statistics included mean and standard deviations of continuous variables and counts for categorical variables. Student's *t-*test was employed for paired and Welcher two-sample t-test for unpaired analysis. Pearson product moment correlation coefficients were used for examining the relationships between cognitive and imaging measures. Significance level was set at *p* < 0.05 (trend-level *p* < 0.1).

## 3 Results

The patients were classified as either amyloid positive (uptake ratio ≥ 1.4) or negative (uptake ratio < 1.4) based on the [^18^F]Flutemetamol composite cortical uptake score at baseline (see Table 1). 9 patients (45%) had a positive and 11 patients (55%) had a negative [^18^F]Flutemetamol scan. There were no statistically significant differences in either age (Table 1, *t*-test *p* = 0.067) or years of education (Table 1, t-test *p* = 0.19) between amyloid positive and negative patients.

### 3.1 Cognition

Cognitive test performances of the participants are presented in Table 2. The mean MMSE at baseline was 26.9 ± 1.7 and no group difference between amyloid positive and negative patients was observed (Table 2). In contrast, the amyloid positive patients exhibited significant decline in MMSE scores in three years (*p* < 0.05, Table 2) while the amyloid negative patients maintained stable MMSE level over time. Consequently, a significant group difference in MMSE was observed after three years (p < 0.05, Table 2). However, decline in MMSE of amyloid positive patients was marginally greater compared to amyloid negative patients' but did not reach statistical significance.

More detailed, domain-specific cognitive tests revealed that the amyloid positive patients had significantly worse episodic memory functions than the amyloid negative patients both at baseline and at follow-up, but the rates of episodic memory decline were commensurate in the two groups (Table 2). Amyloid positive patients exhibited significantly slower processing speed at follow-up, but the difference in rates of decline did not significantly differ between the groups. The amyloid positive patients showed a decline in language functions over time, whereas a test-retest effect (i.e. improvement over time) was observed in the amyloid negative patients (Table 2).

### 3.2 [^18^F]FDG imaging

The mean cortical composite [^18^F]FDG uptake ratio was 1.35 ± 0.09 at baseline and 1.35 ± 0.08 at follow-up (Supplemental Table 1) indicating stable average glucose metabolism over three years in the whole aMCI study group. No significant differences between amyloid positive and negative subgroups were observed in composite cortical [^18^F]FDG uptake ratio at baseline or follow-up (Supplemental Table 1). The absence of generalized group differences in regional [^18^F]FDG uptake ratio and longitudinal change was further supported by region-wise analyses across all Desikan–Killiany cortical regions (*n* = 42; Supplementary Table 1). A statistically significant difference in [^18^F]FDG uptake ratio between amyloid positive and negative groups was observed only in the left cuneus at baseline (Supplemental table 1), such that the amyloid positive group exhibited significantly lower in [^18^F]FDG uptake ratio compared to amyloid negative, and a similar trend was observed in the right cuneus. Consequently, the subsequent [^18^F]FDG uptake ratio analyses were conducted in a pooled sample containing both amyloid positive and negative patients.

**Fig. 1:**
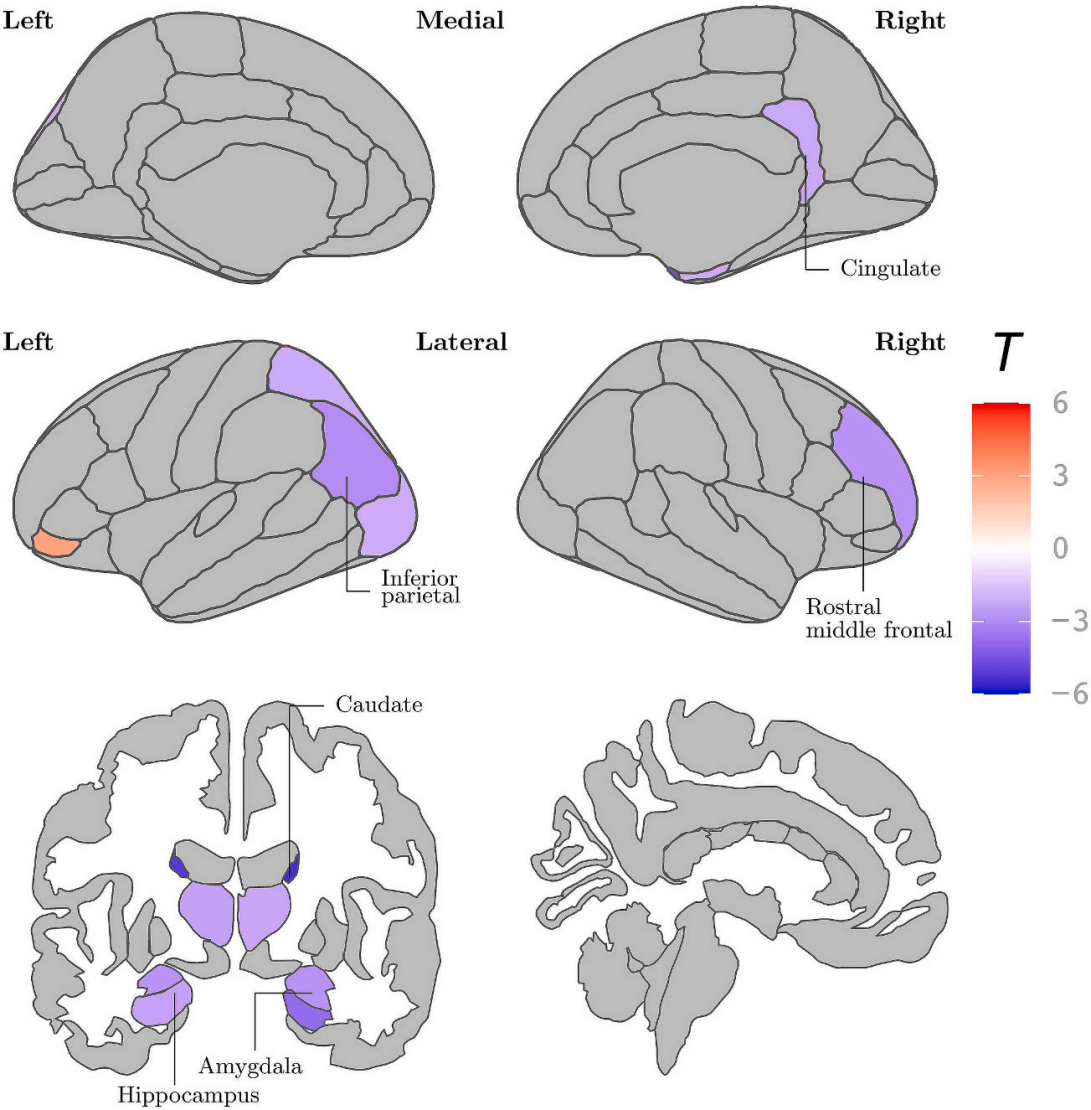
Whole-brain distribution of 3-year change statistics in [^18^F]FDG uptake ratio. T-statistics of paired t-test in the pooled sample over time are shown. Negative t-statistic indicates decline in [^18^F]FDG uptake ratio.

In the pooled sample, whole-brain analysis revealed several regions in the subcortex as well as cortex that exhibited significant [^18^F]FDG decline over three years (Supplemental Table 1, Fig. 1). Specifically, [^18^F]FDG uptake ratio in the left and right caudate and amygdala and right hippocampus declined significantly over three years (*p* < 0.05, Supplemental Table 1, Fig. 1). Cortically, declines were observed in frontoparietal regions and entorhinal cortex (p < 0.05, Supplemental Table 1, Fig. 1). Left pars orbitalis was the only region exhibiting increase in [^18^F]FDG uptake ratio over three years, driven by amyloid positive participants (Supplemental Table 1), even though the group difference was not significant. Furthermore, the composite scores of [^18^F]FDG and [^18^F]Flutemetamol uptake were not significantly correlated (Pearson's correlation coefficient rs = −0.16–0.02, ps > 0.05).

### 3.3 [^18^F]FDG imaging and cognition

**Fig. 2:**
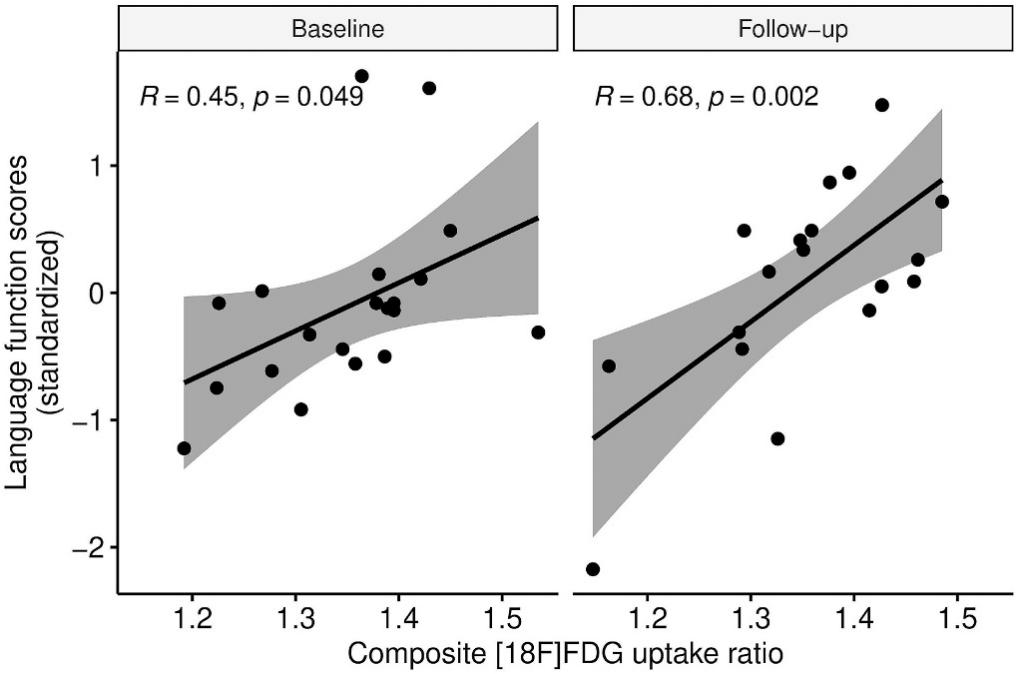
Composite [^18^F]FDG uptake ratio and language functions scores exhibited a significant positive correlation at baseline and follow-up.

Associations between composite [^18^F]FDG uptake ratios and cognitive scores were explored using Pearson's correlation coefficients (Supplemental Table 2). In initial analysis we found no group difference in the association between amyloid positive and negative patients (data not shown), and hence the subsequent analysis were conducted in the entire study group. Statistically significant association was observed with executive function score at baseline and follow-up (*r* = 0.45–0.55, *p* < 0.01, Supplemental Table 2) and with language functions scores at baseline and follow-up (r = 0.45–0.68, p < 0.01, Supplemental Table 2, Fig. 2).

## 4 Discussion

We found no significant differences between the amyloid positive and negative groups in neither [^18^F]FDG uptake at the two measurement time-points nor in the change of [^18^F]FDG uptake over time. By contrast, in terms of cognition, the amyloid positive group exhibited poorer episodic memory at baseline and follow-up and tended to show more cognitive decline over time in most cognitive domains compared to the amyloid negative aMCI patients. Furthermore, we found that [^18^F]FDG uptake was positively associated with executive function and language functions scores across all aMCI patients. However, the cognitive analyses were included primarily to provide clinical context for the longitudinal PET findings rather than to establish a novel cognitive phenotype of aMCI. In the entire cohort, several brain regions exhibited decreases of [^18^F]FDG uptake over time. Specifically, regions typically affected in AD, including medial temporal lobes, frontal, parietal and posterior cortex showed negative longitudinal change of [^18^F]FDG uptake. In addition, [^18^F]FDG uptake in the caudate and amygdala exhibited decreases over time.

The amyloid positive group tended to show lower baseline [^18^F]FDG uptake in several regions, but the difference between the two groups reached statistical significance only in the cuneus. Furthermore, amyloid status was not predictive of longitudinal [^18^F]FDG uptake change. In concordance, in an earlier study Ewers et al. [23] found a trend for a faster [^18^F]FDG decline in amyloid positive aMCI patients in posterior cingulate gyrus and inferior/middle temporal gyrus. Despite having a larger number of aMCI patients than in our study, the study of Ewers et al. [23] observed a statistically non-significant difference. The present results, together with prior findings in amnestic MCI patients hence suggest that [^18^F]FDG uptake is at most weakly negatively affected by positive amyloid status.

Kemppainen et al. [22] showed that compared to normally aged controls, who present with an almost stable trajectory over a 5-year follow-up, the [^18^F]FDG uptake composite score in aMCI patients decreased by 2.0% each year. In another study of up to 3-year follow-up, a 1.3% annual decrease in the [^18^F]FDG uptake composite score in aMCI patients vs. 0.39% decrease in normal controls was found [24]. This is in accordance with other longitudinal [^18^F]FDG PET studies which have reported greater decline in glucose metabolism in aMCI groups compared to normally aged controls [25], [43]. In contrast, we did not find significant decline in the [^18^F]FDG composite score over time in our aMCI patients. This might be due to the small number of patients or differences in brain areas included in the composite score. However, we found significant decline in [^18^F]FDG uptake for instance in certain subcortical areas, such as hippocampus and entorhinal cortex not included in composite score. The composition of the FDG composite ROI may influence sensitivity to subtle or regionally restricted longitudinal metabolic changes. However, the present analyses were not limited to the composite ROI: group differences were also examined separately across all Desikan–Killiany cortical regions. These supplementary region-wise analyses did not reveal a consistent pattern suggesting that the absence of group differences was driven by dilution within the composite ROI. Thus, although alternative AD-sensitive meta-ROIs may be useful in larger cohorts, it is unlikely that modest changes in the composition of the present composite ROI would substantially alter the overall interpretation of the current findings.

We showed a decline in cognitive scores over the 3-year follow-up period in amyloid positive aMCI patients in all cognitive domains, as well as in the screening test of general cognitive functioning (MMSE). The decline reached significance only in the MMSE (which was also shown in our earlier study [10]. In the amyloid negative group, functioning in all cognitive domains remained essentially stable, with a slight test-retest effect (improvement) being observed in language functions. The absence of statistically significant difference in cognitive change scores may partly be attributed to the relatively small sample size in the present study, limiting the statistical power to detect differences. Our results are in accordance with an earlier study [23] which also showed a faster decline in cognitive tests in amyloid positive patients compared to negative ones in aMCI patients in a 2-year follow-up. They also found a statistically significant difference between amyloid positive and negative patients in a bigger patient population of 229 aMCI patients in the decline on the ADAS-Cog and delayed recall scores. Furthermore, a greater ADAS-Cog decline in in amyloid positive late aMCI patients compared to negative ones has also been found [44]. On the contrary, Dubois et al. [26] found no differences between amyloid positive and negative patients in any of the most used cognitive tests in a 2-year follow-up. Different patient populations can be one reason for the discrepancies between studies. Dubois et al. [26] studied patients with subjective memory impairment with MMSE ≥27, and amnestic MCI patients were not separated. In our study as well as in the study of Ewers et al. [23], patients were amnestic MCI patients with an MMSE-score between 24 and 30. It has been suggested that patients with amnestic MCI are more likely to clinically progress to AD than patients with non-amnestic MCI [29], [45], [46], [47]. In non-amnestic MCI patients, the pathology of the underlying disease can be more often other than AD.

In all aMCI patients we found a significant decline in [^18^F]FDG uptake in the left and right caudate, amygdala and right hippocampus as well as in frontoparietal regions and entorhinal cortex. This is in accordance with earlier studies [22], [43], [48]. We found a positive association between [^18^F]FDG uptake and cognition in the entire group of aMCI patients, but no difference in this association between amyloid positive and negative patient groups. In earlier longitudinal studies, the decline in [^18^F]FDG uptake has been associated with cognitive decline in aMCI patients [24], [25], [43] and the change in [^18^F]FDG scores correlated significantly with decline in MMSE scores both in stable MCI patient and in prodromal AD patient groups [27]. This suggests that brain glucose metabolism is independent of amyloid pathology, and refers more probably on brain degenerative processes.

Several limitations should be considered when interpreting the present findings. First, the sample size was modest, and the cohort was further divided into amyloid-positive and amyloid-negative subgroups. This limited the statistical power to detect subtle group differences and precluded reliable subgroup analyses. In particular, the sample included more men than women, and sex-stratified analyses were therefore not performed to avoid overinterpretation of unstable estimates. Second, ApoE genotype data were not available, limiting our ability to assess whether genetic risk modified longitudinal metabolic or cognitive trajectories. In addition, tau biomarkers were not available, and the diagnostic terminology therefore refers to clinically probable AD rather than full biological AD classification according to the ATN framework. These limitations notwithstanding, the study was conducted at a single imaging site, included a clinically homogeneous group of patients with amnestic MCI, and used longitudinal cognitive, MRI, [18F]FDG PET, and [18F]Flutemetamol PET assessments in the same individuals. Future studies should validate the present findings in larger longitudinal cohorts with balanced sex distributions, ApoE characterization, and broader biomarker assessment.

## 5 Conclusions

In this study, we found greater cognitive decline over time in amyloid positive patients. However, [^18^F]FDG uptake or change in [^18^F]FDG uptake over time did not generally differ between the amyloid positive and negative groups. However, several brain regions typically affected in AD, including medial temporal lobes, frontal, parietal and posterior cortex showed decreases of [^18^F]FDG uptake over time in the whole aMCI group. Moreover, there was a positive association between [^18^F]FDG uptake and cognition in the overall group of aMCI patients, but no difference in this association between amyloid positive and negative patient groups. Furthermore, the composite scores of [18F]FDG and [18F]Flutemetamol uptake were not significantly correlated. These findings suggest that brain glucose metabolism in aMCI is independent of amyloid pathology, and probably reflects other neurodegenerative processes. Future studies in larger longitudinal cohorts, ideally including balanced sex distributions, ApoE genotyping, and tau biomarker assessment, are needed to confirm these findings.

## Registration

This was an academic study with no drug or other intervention and therefore the study was not registered in any registries.

## CRediT authorship contribution statement

**Elina Rauhala:** Writing – review & editing, Writing – original draft, Project administration, Investigation, Conceptualization. **Jarkko Johansson:** Writing – review & editing, Writing – original draft, Visualization, Validation, Software, Methodology, Investigation, Formal analysis, Data curation, Conceptualization. **Mira Karrasch:** Writing – review & editing, Writing – original draft, Validation, Data curation, Conceptualization. **Olli Eskola:** Writing – review & editing, Writing – original draft, Resources. **Tuula Tolvanen:** Writing – review & editing, Writing – original draft, Validation, Resources, Data curation. **Johan Rajander:** Writing – review & editing, Writing – original draft, Resources. **Juha O. Rinne:** Writing – review & editing, Writing – original draft, Validation, Supervision, Resources, Project administration, Investigation, Funding acquisition, Data curation, Conceptualization.

## Consent to participate

Informed consent was obtained from all individual participants included in the study.

## Consent to publish

Informed consent was obtained from all individual participants included in the study, including that imaging data can be used in research purposes and can be published in international scientific journals without possibility to identify individual participant.

## Ethics approval

This study was performed in line with the principles of the Declaration of Helsinki. Approval was granted by the Ethics Committee of Hospital District of Southwest Finland on the 15th of February 2011.

## Funding

This work was financially supported by the 10.13039/501100003125Finnish Cultural Foundation, the 10.13039/501100007417Paulo Foundation, the Finnish Medicine Foundation, the Maire Taponen Foundation, the 10.13039/100010129Maud Kuistila Memorial Foundation, Finnish Academy (project # 310962), the Sigrid Juselius Foundation and Finnish State Research Funding (VTR).

## Declaration of competing interest

The authors declare the following financial interests/personal relationships which may be considered as potential competing interests: Juha Rinne reports financial support was provided by Finnish Medicine Foundation. Juha Rinne reports a relationship with Clinical Research Services Turku that includes: consulting or advisory. Juha Rinne reports a relationship with Novo Nordisk that includes: consulting or advisory. Juha Rinne reports a relationship with BioArctic AB that includes: consulting or advisory. Juha Rinne reports a relationship with Eli Lilly and Novartis that includes: consulting or advisory. Juha Rinne reports a relationship with Lundbeck LLC that includes: consulting or advisory. If there are other authors, they declare that they have no known competing financial interests or personal relationships that could have appeared to influence the work reported in this paper.

## Data Availability

Due to the consent given by study participants and the high degree of identifiability, data cannot be made publicly available. Pseudonymised data may be shared with authorized researchers, upon researcher's reasonable request, who have IRB/ethics approval and an institutionally approved study plan.

## References

[1] Greenberg J.H., Reivich M., Alavi A., Hand P., Rosenquist A., Rintelmann W. (1981). Metabolic mapping of functional activity in human subjects with the [18F]fluorodeoxyglucose technique. Science.

[2] Edison P., Archer H., Hinz R., Hammers A., Pavese N., Tai Y.F. (2007). Amyloid, Hypometabolism, and Cognition in Alzheimer Disease An [11C]PIB and [18F]FDG PET Study. http://www.neurology.org.

[3] Mosconi L., Mistur R., Switalski R., Tsui W.H., Glodzik L., Li Y. (2009). FDG-PET changes in brain glucose metabolism from normal cognition to pathologically verified Alzheimer’s disease. Eur J Nucl Med Mol Imaging.

[4] Nordberg A., Rinne J.O., Kadir A., Långström B. (2010). The use of PET in Alzheimer disease. Nat Rev Neurol.

[5] Kadir A., Almkvist O., Forsberg A., Wall A., Engler H., Långström B. (2012). Dynamic changes in PET amyloid and FDG imaging at different stages of Alzheimer’s disease. Neurobiol Aging.

[6] Li Y., Rinne J.O., Mosconi L., Pirraglia E., Rusinek H., Desanti S. (2008). Regional analysis of FDG and PIB-PET images in normal aging, mild cognitive impairment, and Alzheimer’s disease. Eur J Nucl Med Mol Imaging.

[7] Mosconi L., Perani D., Sorbi S., Herholz K., Nacmias B., Holthoff V. (2004). MCI conversion to dementia and the APOE genotype: a prediction study with FDG-PET. Neurology.

[8] Ossenkoppele R., Tolboom N., Foster-Dingley J.C., Adriaanse S.F., Boellaard R., Yaqub M. (2012). Longitudinal imaging of Alzheimer pathology using [ 11C]PIB, [ 18F]FDDNP and [ 18F]FDG PET. Eur J Nucl Med Mol Imaging.

[9] Hatashita S., Wakebe D., Kikuchi Y., Ichijo A. (2019). Longitudinal assessment of amyloid-β deposition by [18F]-Flutemetamol PET imaging compared with [11C]-PIB across the spectrum of Alzheimer’s disease. Front Aging Neurosci.

[10] Rauhala E., Johansson J., Karrasch M., Eskola O., Tolvanen T., Parkkola R. (2022). Change in brain amyloid load and cognition in patients with amnestic mild cognitive impairment: a 3-year follow-up study. EJNMMI Res.

[11] Wolk D.A., Sadowsky C., Safirstein B., Rinne J.O., Duara R., Perry R. (2018). Use of flutemetamol F18-labeled positron emission tomography and other biomarkers to assess risk of clinical progression in patients with amnestic mild cognitive impairment. JAMA Neurol.

[12] Brück A., Virta J.R., Koivunen J., Koikkalainen J., Scheinin N.M., Helenius H. (2013). PIB, [18F]FDG and MR imaging in patients with mild cognitive impairment. Eur J Nucl Med Mol Imaging.

[13] Jack C.R., Knopman D.S., Jagust W.J., Petersen R.C., Weiner M.W., Aisen P.S. (2013). Tracking pathophysiological processes in Alzheimer’s disease: an updated hypothetical model of dynamic biomarkers. Lancet Neurol.

[14] Koivunen J., Scheinin N., Virta J.R., Aalto S., Vahlberg T., Någren K. (2011). Amyloid PET imaging in patients with mild cognitive impairment: a 2-year follow-up study. Neurology.

[15] Villemagne V.L., Burnham S., Bourgeat P., Brown B., Ellis K.A., Salvado O. (2013). Amyloid β deposition, neurodegeneration, and cognitive decline in sporadic Alzheimer’s disease: a prospective cohort study. Lancet Neurol.

[16] Minoshima S., Giordani B., Berent S., Frey K.A., Foster N.L., Kuhl D.E. (1997). Metabolic reduction in the posterior cingulate cortex in very early Alzheimer’s disease. Ann Neurol.

[17] Blazhenets G., Ma Y., Sörensen A., Rücker G., Schiller F., Eidelberg D. (2019). Principal components analysis of brain metabolism predicts development of Alzheimer dementia. J Nucl Med.

[18] Mosconi L., Tsui W.-H., De Santi S., Li J., Rusinek H., Convit A. (2005). Reduced hippocampal metabolism in MCI and AD: automated FDG-PET image analysis. Neurology.

[19] Ashraf A., Fan Z., Brooks D.J., Edison P. (2015). Cortical hypermetabolism in MCI subjects: a compensatory mechanism?. Eur J Nucl Med Mol Imaging.

[20] Cohen A.D., Price J.C., Weissfeld L.A., James J., Rosario B.L., Bi W. (2009). Basal cerebral metabolism may modulate the cognitive effects of Abeta in mild cognitive impairment: an example of brain reserve. J Neurosci.

[21] Rubinski A., Franzmeier N., Neitzel J., Ewers M. (2020). FDG-PET hypermetabolism is associated with higher tau-PET in mild cognitive impairment at low amyloid-PET levels. Alzheimer’s Res Ther.

[22] Kemppainen N., Joutsa J., Johansson J., Scheinin N.M., Någren K., Rokka J., Barthel H. (2015). J Alzheimers Dis [internet].

[23] Ewers M., Insel P., Jagust W.J., Shaw L., Trojanowski J.J.Q., Aisen P. (2012). CSF biomarker and PIB-PET-derived beta-amyloid signature predicts metabolic, gray matter, and cognitive changes in nondemented subjects. Cereb Cortex.

[24] Landau S.M., Harvey D., Madison C.M., Koeppe R.A., Reiman E.M., Foster N.L. (2011). Associations between cognitive, functional, and FDG-PET measures of decline in AD and MCI. Neurobiol Aging.

[25] Shokouhi S., Claassen D., Kang H., Ding Z., Rogers B., Mishra A. (2013). Longitudinal progression of cognitive decline correlates with changes in the spatial pattern of brain 18F-FDG PET. J Nucl Med.

[26] Dubois B., Epelbaum S., Nyasse F., Bakardjian H., Gagliardi G., Uspenskaya O. (2018). Cognitive and neuroimaging features and brain β-amyloidosis in individuals at risk of Alzheimer’s disease (INSIGHT-preAD): a longitudinal observational study. Lancet Neurol.

[27] Beheshti I., Geddert N., Perron J., Gupta V., Albensi B.C., Ko J.H. (2022). Monitoring Alzheimer’s disease progression in mild cognitive impairment stage using machine learning-based FDG-PET classification methods. J Alzheimer’s Dis.

[28] Bakhtiari A., Benedek K., Law I., Fagerlund B., Mortensen E.L., Osler M. (2024). Early cerebral amyloid-β accumulation and hypermetabolism are associated with subtle cognitive deficits before accelerated cerebral atrophy. Geroscience.

[29] Petersen R.C. (2004). Mild cognitive impairment as a diagnostic entity. J Intern Med.

[30] Jack C.R., Bennett D.A., Blennow K., Carrillo M.C., Dunn B., Haeberlein S.B. (2018). NIA-AA research framework: toward a biological definition of Alzheimer’s disease. Alzheimers Dement.

[31] Duara R., Loewenstein D.A., Shen Q., Barker W., Potter E., Varon D. (2013). Amyloid positron emission tomography with 18F-flutemetamol and structural magnetic resonance imaging in the classification of mild cognitive impairment and Alzheimer’s disease. Alzheimers Dement.

[32] Wechsler D. (1981).

[33] Wechsler D. (1996).

[34] Benton A.L., Hamsher K. (1976).

[35] Reitan R.M. (1958). Validity of the trail making test as an indicator of organic brain damage. Percept Mot Skills.

[36] Welsh K.A., Butters N., Mohs R.C., Beekly D., Edland S., Fillenbaum G. (1994). The consortium to establish a registry for Alzheimer’s disease (CERAD). Part V. A normative study of the neuropsychological battery. Neurology.

[37] Lezak M.D., Howieson D.B., Bigler E.D., Tranel D. (2012).

[38] Toppala S., Ekblad L.L., Lötjönen J., Helin S., Hurme S., Johansson J. (2019). Midlife insulin resistance as a predictor for late-life cognitive function and cerebrovascular lesions. J Alzheimer’s Dis.

[39] Bettinardi V., Presotto L., Rapisarda E., Picchio M., Gianolli L., Gilardi M.C. (2011). Physical performance of the new hybrid PETCT Discovery-690. Med Phys.

[40] Desikan R.S., Ségonne F., Fischl B., Quinn B.T., Dickerson B.C., Blacker D. (2006). An automated labeling system for subdividing the human cerebral cortex on MRI scans into gyral based regions of interest. Neuroimage.

[41] Scheinin N.M., Aalto S., Koikkalainen J., Lötjönen D.J., Karrasch D.M., Kemppainen N. (2009). Follow-up of [ 11 C]PIB uptake and brain volume in patients with Alzheimer disease and controls. http://www.neurology.org.

[42] Jagust W., Reed B., Mungas D., Ellis W., DeCarli C. (2007). What does fluorodeoxyglucose PET imaging add to a clinical diagnosis of dementia?. Neurology.

[43] Chen K., Langbaum J.B.S., Fleisher A.S., Ayutyanont N., Reschke C., Lee W. (2010). Twelve-month metabolic declines in probable Alzheimer’s disease and amnestic mild cognitive impairment assessed using an empirically pre-defined statistical region-of-interest: findings from the Alzheimer’s disease neuroimaging initiative. Neuroimage.

[44] Landau S.M., Mintun M.A., Joshi A.D., Koeppe R.A., Petersen R.C., Aisen P.S. (2012). Amyloid deposition, hypometabolism, and longitudinal cognitive decline. Ann Neurol.

[45] Pike K.E., Savage G., Villemagne V.L., Ng S., Moss S.A., Maruff P. (2007). β-Amyloid imaging and memory in non-demented individuals: evidence for preclinical Alzheimer’s disease. Brain.

[46] Ritchie L.J., Tuokko H. (2010). Patterns of cognitive decline, conversion rates, and predictive validity for 3 models of MCI. Am J Alzheimers Dis Other Dement.

[47] Vos S.J.B., van Rossum I.A., Verhey F., Knol D.L., Soininen H., Wahlund L.-O. (2013). Prediction of Alzheimer disease in subjects with amnestic and nonamnestic MCI. Neurology.

[48] Fouquet M., Desgranges B., Landeau B., Duchesnay E., Mézenge F., de la Sayette V. (2009). Longitudinal brain metabolic changes from amnestic mild cognitive impairment to Alzheimer’s disease. Brain.

