# Supplementary material for: Change in brain glucose metabolism and cognition in amnestic MCI patients in relation to amyloid accumulation: A 3-year follow-up study
Source: Aging Brain. 2026 Aug 6;10:100165. doi: 10.1016/j.nbas.2026.100165 (PMC13475694; doi:10.1016/j.nbas.2026.100165)
Supplement: Supplementary file 1 [file mmc1.docx]

**Supplemental material**

**Supplemental Table 1**: [^18^F]FDG PET imaging results stratified by test time and amyloid status. Mean (±SD) [^18^F]FDG uptake ratio is shown in each patient group and time point. Paired t-tests were conducted to assess the significance of change over time (delta), and two-sample t-tests were conducted to assess the group difference in level and change across the amyloid positive and negative patients. Results for composite score (see Methods) and separate regions with significant effect are shown. * p < 0.05, ** p < 0.01.

|  | All | Amyloid Positive | Amyloid Negative | Group Difference |
| --- | --- | --- | --- | --- |
| Composite score baseline | 1.35±0.08 (N=20) | 1.34±0.08 (N=9) | 1.36±0.09 (N=11) | t(18)=-0.46 |
| Composite score followup | 1.35±0.09 (N=18) | 1.33±0.11 (N=7) | 1.36±0.09 (N=11) | t(11)=-0.65 |
| Composite score delta | -0.00±0.06 | -0.01±0.05 | 0.00±0.07 | t(15)=-0.46 |
| Amygdala Left baseline | 0.98±0.08 (N=20) | 0.96±0.07 (N=9) | 0.99±0.09 (N=11) | t(18)=-0.97 |
| Amygdala Left followup | 0.96±0.12 (N=18) | 0.93±0.12 (N=7) | 0.98±0.12 (N=11) | t(13)=-0.74 |
| Amygdala Left delta | **-0.02±0.04*** | -0.03±0.05 | -0.02±0.04 | t(10)=-0.88 |
| Amygdala Right baseline | 0.98±0.09 (N=20) | 0.96±0.08 (N=9) | 0.99±0.09 (N=11) | t(18)=-0.87 |
| Amygdala Right followup | 0.97±0.10 (N=18) | 0.94±0.08 (N=7) | 0.98±0.11 (N=11) | t(15)=-0.80 |
| Amygdala Right delta | **-0.02±0.04*** | -0.04±0.04. | -0.01±0.04 | t(12)=-1.24 |
| Caudate Left baseline | 0.92±0.31 (N=20) | 0.90±0.23 (N=9) | 0.94±0.37 (N=11) | t(17)=-0.36 |
| Caudate Left followup | 0.83±0.32 (N=18) | 0.82±0.32 (N=7) | 0.84±0.34 (N=11) | t(14)=-0.13 |
| Caudate Left delta | **-0.11±0.09***** | **-0.12±0.12*** | **-0.10±0.07**** | t(9)=-0.39 |
| Caudate Right baseline | 0.96±0.30 (N=20) | 0.91±0.20 (N=9) | 1.00±0.37 (N=11) | t(16)=-0.73 |
| Caudate Right followup | 0.88±0.31 (N=18) | 0.84±0.26 (N=7) | 0.91±0.35 (N=11) | t(15)=-0.51 |
| Caudate Right delta | **-0.10±0.07***** | **-0.11±0.10*** | **-0.09±0.05***** | t(8)=-0.35 |
| Hippocampus Left baseline | 0.97±0.09 (N=20) | 0.95±0.07 (N=9) | 0.99±0.10 (N=11) | t(18)=-1.01 |
| Hippocampus Left followup | 0.96±0.12 (N=18) | 0.94±0.12 (N=7) | 0.97±0.13 (N=11) | t(14)=-0.56 |
| Hippocampus Left delta | -0.02±0.04. | -0.03±0.05 | -0.02±0.04 | t(10)=-0.43 |
| Hippocampus Right baseline | 0.97±0.09 (N=20) | 0.94±0.08 (N=9) | 1.00±0.10 (N=11) | t(18)=-1.35 |
| Hippocampus Right followup | 0.95±0.12 (N=18) | 0.90±0.12 (N=7) | 0.98±0.12 (N=11) | t(14)=-1.29 |
| Hippocampus Right delta | **-0.03±0.04**** | **-0.05±0.04*** | -0.02±0.04. | t(12)=-1.67 |
| Cuneus Left baseline | 1.44±0.13 (N=20) | 1.36±0.13 (N=9) | 1.50±0.10 (N=11) | **t(16)=-2.75*** |
| Cuneus Left followup | 1.42±0.14 (N=18) | 1.35±0.17 (N=7) | 1.46±0.10 (N=11) | t(9)=-1.61 |
| Cuneus Left delta | -0.03±0.08 | -0.01±0.08 | -0.04±0.08 | t(13)=0.76 |
| Cuneus Right baseline | 1.48±0.11 (N=20) | 1.43±0.10 (N=9) | 1.52±0.10 (N=11) | t(17)=-2.09. |
| Cuneus Right followup | 1.45±0.12 (N=18) | 1.40±0.12 (N=7) | 1.49±0.11 (N=11) | t(12)=-1.64 |
| Cuneus Right delta | -0.03±0.09 | -0.02±0.10 | -0.03±0.09 | t(12)=0.22 |
| Entorhinal Left baseline | 0.88±0.08 (N=20) | 0.86±0.08 (N=9) | 0.89±0.08 (N=11) | t(18)=-1.02 |
| Entorhinal Left followup | 0.86±0.10 (N=18) | 0.83±0.11 (N=7) | 0.87±0.09 (N=11) | t(11)=-0.96 |
| Entorhinal Left delta | **-0.02±0.04*** | -0.02±0.04 | -0.02±0.04 | t(13)=-0.09 |
| Entorhinal Right baseline | 0.92±0.09 (N=20) | 0.92±0.06 (N=9) | 0.92±0.10 (N=11) | t(16)=-0.01 |
| Entorhinal Right followup | 0.90±0.11 (N=18) | 0.88±0.07 (N=7) | 0.90±0.13 (N=11) | t(16)=-0.49 |
| Entorhinal Right delta | **-0.02±0.03*** | -0.02±0.02. | -0.02±0.04 | t(16)=-0.28 |
| Frontalpole Left baseline | 1.11±0.16 (N=20) | 1.12±0.16 (N=9) | 1.10±0.17 (N=11) | t(17)=0.24 |
| Frontalpole Left followup | 1.01±0.11 (N=18) | 1.00±0.12 (N=7) | 1.02±0.10 (N=11) | t(12)=-0.34 |
| Frontalpole Left delta | **-0.08±0.10**** | -0.09±0.12. | **-0.08±0.10*** | t(11)=-0.18 |
| Frontalpole Right baseline | 1.21±0.14 (N=20) | 1.23±0.12 (N=9) | 1.20±0.16 (N=11) | t(18)=0.50 |
| Frontalpole Right followup | 1.13±0.10 (N=18) | 1.13±0.09 (N=7) | 1.14±0.10 (N=11) | t(14)=-0.25 |
| Frontalpole Right delta | **-0.07±0.10**** | -0.09±0.13 | -0.06±0.09. | t(9)=-0.64 |
| Inferiorparietal Left baseline | 1.16±0.11 (N=20) | 1.12±0.12 (N=9) | 1.19±0.10 (N=11) | t(15)=-1.46 |
| Inferiorparietal Left followup | 1.12±0.14 (N=18) | 1.06±0.18 (N=7) | 1.16±0.10 (N=11) | t(8)=-1.41 |
| Inferiorparietal Left delta | **-0.04±0.06*** | -0.06±0.07. | -0.03±0.06 | t(11)=-1.08 |
| Inferiorparietal Right baseline | 1.13±0.10 (N=20) | 1.11±0.09 (N=9) | 1.15±0.11 (N=11) | t(18)=-0.98 |
| Inferiorparietal Right followup | 1.11±0.14 (N=18) | 1.05±0.15 (N=7) | 1.14±0.13 (N=11) | t(11)=-1.36 |
| Inferiorparietal Right delta | -0.03±0.08. | -0.06±0.08. | -0.01±0.07 | t(11)=-1.45 |
| Lateraloccipital Left baseline | 1.17±0.13 (N=20) | 1.11±0.14 (N=9) | 1.21±0.10 (N=11) | t(14)=-1.75 |
| Lateraloccipital Left followup | 1.15±0.14 (N=18) | 1.12±0.18 (N=7) | 1.17±0.11 (N=11) | t(9)=-0.68 |
| Lateraloccipital Left delta | **-0.03±0.06*** | -0.02±0.06 | **-0.04±0.05*** | t(11)=0.66 |
| Lateraloccipital Right baseline | 1.18±0.10 (N=20) | 1.16±0.11 (N=9) | 1.20±0.08 (N=11) | t(14)=-0.83 |
| Lateraloccipital Right followup | 1.17±0.10 (N=18) | 1.16±0.13 (N=7) | 1.18±0.09 (N=11) | t(10)=-0.31 |
| Lateraloccipital Right delta | -0.02±0.07 | -0.03±0.10 | -0.02±0.04 | t(8)=-0.13 |
| Parsorbitalis Left baseline | 1.27±0.17 (N=20) | 1.29±0.13 (N=9) | 1.25±0.20 (N=11) | t(17)=0.55 |
| Parsorbitalis Left followup | 1.31±0.15 (N=18) | 1.35±0.16 (N=7) | 1.29±0.15 (N=11) | t(12)=0.80 |
| Parsorbitalis Left delta | **0.05±0.08*** | **0.07±0.07*** | 0.04±0.09 | t(15)=0.82 |
| Parsorbitalis Right baseline | 1.20±0.17 (N=20) | 1.20±0.11 (N=9) | 1.19±0.21 (N=11) | t(15)=0.17 |
| Parsorbitalis Right followup | 1.21±0.17 (N=18) | 1.21±0.13 (N=7) | 1.21±0.19 (N=11) | t(16)=0.00 |
| Parsorbitalis Right delta | 0.02±0.06 | 0.02±0.04 | 0.02±0.08 | t(16)=-0.25 |
| Rostralmiddlefrontal Left baseline | 1.29±0.14 (N=20) | 1.27±0.11 (N=9) | 1.30±0.16 (N=11) | t(18)=-0.48 |
| Rostralmiddlefrontal Left followup | 1.27±0.13 (N=18) | 1.22±0.14 (N=7) | 1.30±0.12 (N=11) | t(12)=-1.24 |
| Rostralmiddlefrontal Left delta | -0.02±0.08 | -0.04±0.05. | -0.00±0.10 | t(16)=-1.04 |
| Rostralmiddlefrontal Right baseline | 1.29±0.15 (N=20) | 1.29±0.12 (N=9) | 1.29±0.17 (N=11) | t(18)=0.07 |
| Rostralmiddlefrontal Right followup | 1.24±0.14 (N=18) | 1.21±0.15 (N=7) | 1.26±0.14 (N=11) | t(12)=-0.69 |
| Rostralmiddlefrontal Right delta | **-0.04±0.07*** | **-0.07±0.06*** | -0.03±0.08 | t(15)=-1.28 |
| Superiorparietal Left baseline | 1.15±0.12 (N=20) | 1.11±0.11 (N=9) | 1.19±0.12 (N=11) | t(18)=-1.56 |
| Superiorparietal Left followup | 1.12±0.12 (N=18) | 1.05±0.13 (N=7) | 1.16±0.10 (N=11) | t(11)=-1.97. |
| Superiorparietal Left delta | **-0.04±0.07*** | **-0.06±0.06*** | -0.03±0.08 | t(16)=-0.94 |
| Superiorparietal Right baseline | 1.11±0.10 (N=20) | 1.06±0.09 (N=9) | 1.15±0.10 (N=11) | t(18)=-1.96. |
| Superiorparietal Right followup | 1.07±0.12 (N=18) | 0.99±0.13 (N=7) | 1.12±0.09 (N=11) | t(10)=-2.20. |
| Superiorparietal Right delta | -0.04±0.08. | -0.06±0.08. | -0.02±0.09 | t(14)=-0.95 |
| Temporalpole Left baseline | 0.84±0.09 (N=20) | 0.84±0.09 (N=9) | 0.84±0.10 (N=11) | t(18)=0.11 |
| Temporalpole Left followup | 0.80±0.12 (N=18) | 0.80±0.12 (N=7) | 0.80±0.12 (N=11) | t(13)=-0.00 |
| Temporalpole Left delta | **-0.04±0.05**** | -0.03±0.06 | **-0.04±0.05*** | t(12)=0.26 |
| Temporalpole Right baseline | 0.84±0.11 (N=20) | 0.86±0.09 (N=9) | 0.82±0.11 (N=11) | t(18)=1.02 |
| Temporalpole Right followup | 0.79±0.12 (N=18) | 0.80±0.09 (N=7) | 0.77±0.14 (N=11) | t(16)=0.55 |
| Temporalpole Right delta | **-0.04±0.04***** | **-0.03±0.02**** | **-0.04±0.04**** | t(16)=0.61 |

**Supplemental Table 2**: Association between composite [^18^F]FDG score and cognitive scores.

|  | Pearson's correlation with composite FDG uptake ratio |
| --- | --- |
| MMSE baseline | r=0.16 (N=20) |
| MMSE followup | r=0.08 (N=18) |
| MMSE delta | r=-0.42. |
| Episodic Memory baseline | r=-0.02 (N=20) |
| Episodic Memory followup | r=0.20 (N=18) |
| Episodic Memory delta | r=0.08 |
| Executive function baseline | **r=0.45* (N=20)** |
| Executive function baseline followup | **r=0.54* (N=20)** |
| Executive function baseline delta | r=-0.11 |
| Processing Speed baseline | r=0.02 (N=20) |
| Processing Speed followup | r=0.19 (N=20) |
| Processing Speed delta | r=0.18 |
| Language Functions baseline | **r=0.45* (N=20)** |
| Language Functions followup | **r=0.68** (N=18)** |
| Language Functions delta | r=0.03 |
|  |  |
|  |  |
|  |  |
